# Targeting the PI3K/AKT/mTOR pathway offer a promising therapeutic strategy for cholangiocarcinoma patients with high doublecortin-like kinase 1 expression

**DOI:** 10.1007/s00432-024-05875-3

**Published:** 2024-07-09

**Authors:** Ziwei Liang, Yang Ge, Jianjian Li, Yunting Bai, Zeru Xiao, Rui Yan, Guangyu An, Donglei Zhang

**Affiliations:** 1 Department of Oncology, Beijing Chao Yang Hospital, Capital Medical University, 8 Gongren Tiyuchang Nanlu Road, Chaoyang Dist., Beijing, 100020 China; 2 Department of Basic Medical Sciences, School of Medicine, Tsinghua-Peking Center for Life Sciences, Institute for Immunology, Tsinghua University, Beijing, 100084 China; 3 Department of Gastroenterology, Beijing Chao Yang Hospital, Capital Medical University, 8 Gongren Tiyuchang Nanlu Road, Chaoyang Dist., Beijing, 100020 China

**Keywords:** Cholangiocarcinoma, DCLK1, Epithelial-mesenchymal transformation, PI3K/AKT/mTOR pathway

## Abstract

**Background:**

Cholangiocarcinoma (CCA), characterized by high heterogeneity and extreme malignancy, has a poor prognosis. Doublecortin-like kinase 1 (DCLK1) promotes a variety of malignant cancers in their progression. Targeting DCLK1 or its associated regulatory pathways can prevent the generation and deterioration of several malignancies. However, the role of DCLK1 in CCA progression and its molecular mechanisms remain unknown. Therefore, we aimed to investigate whether and how DCLK1 contributes to CCA progression.

**Methods:**

The expression of DCLK1 in CCA patients was detected using Immunohistochemistry (IHC). We established DCLK1 knockout and DCLK1 overexpression cell lines for Colony Formation Assay and Transwell experiments to explore the tumor-promoting role of DCLK1. RT-PCR, Western blot and multiple fluorescent staining were used to assess the association between DCLK1 and epithelial–mesenchymal transition (EMT) markers. RNA sequencing and bioinformatics analysis were performed to identify the underlying mechanisms by which DCLK1 regulates CCA progression and the EMT program.

**Results:**

DCLK1 was overexpressed in CCA tissues and was associated with poor prognosis. DCLK1 overexpression facilitated CCA cell invasion, migration, and proliferation, whereas DCLK1 knockdown reversed the malignant tendencies of CCA cells, which had been confirmed both in vivo and in vitro. Furthermore, we demonstrated that DCLK1 was substantially linked to the advancement of the EMT program, which included the overexpression of mesenchymal markers and the downregulation of epithelial markers. For the underlying mechanism, we proposed that the PI3K/AKT/mTOR pathway is the key process for the role of DCLK1 in tumor progression and the occurrence of the EMT program. When administered with LY294002, an inhibitor of the PI3K/AKT/mTOR pathway, the tumor’s ability to proliferate, migrate, and invade was greatly suppressed, and the EMT process was generally reversed.

**Conclusions:**

DCLK1 facilitates the malignant biological behavior of CCA cells through the PI3K/AKT/mTOR pathway. In individuals with cholangiocarcinoma who express DCLK1 at high levels, inhibitors of the PI3K/AKT/mTOR signaling pathway may be an effective therapeutic approach.

**Supplementary Information:**

The online version contains supplementary material available at 10.1007/s00432-024-05875-3.

## Introduction

Cholangiocarcinoma (CCA), accounting for 10–15% of primary liver cancer cases, is one of the most fatal neoplasms with a high lethality rate worldwide. Based on their different anatomic origin within the biliary tree, CCA is primarily classified into two categories: extrahepatic cholangiocarcinoma (eCCA) and intrahepatic cholangiocarcinoma (iCCA) (Kendall et al. 2019; Sarcognato et al. 2021). Cholangiocarcinoma patients are often diagnosed at advanced stages due to early-stage silent symptoms, leading to a poor prognosis. Despite a stable incidence, CCA remains highly lethal, ranking in the top 10 for cancer mortality, with a 5-year survival rate of only 10% (Siegel, et al. 2023; Banales et al. 2020; Vithayathil and Khan 2022). For patients with early-stage CCA, liver resection is always the best treatment option, while in other cases, systemic chemotherapy with Gemcitabine and Cisplatin is the first-line therapy (Rizvi and Gores 2013). Even with emerging targeted therapies and immune checkpoint inhibitors, the cholangiocarcinoma prognosis remains daunting (Elvevi et al. 2022). Therefore, it is critical to explore pivotal biomarkers and specific molecular mechanisms in CCA progression to improve prognosis.

Doublecortin-like kinase 1 (DCLK1), a notable member of the protein kinase superfamily and the doublecortin family, has been implicated in driving malignant progression and epithelial-mesenchymal transition (EMT) in numerous tumors (Li and Bellows 2013; Patel et al. 2016; Ye et al. 2024). In the last decades, it has been discovered that certain malignant neoplasms have increased expression of DCLK1, including pancreatic cancer (Li et al. 2018), Esophageal squamous cell carcinoma (ESCC) (Ge et al. 2021), Renal clear cell carcinoma (RCC) (Ge et al. 2018), stomach adenocarcinoma (STAD) and colorectal cancer (CRC) (Wu et al. 2020; Gao et al. 2016). In addition, several studies suggested that the overexpression of DCLK1 is significantly associated with distant metastasis, poor prognosis, and drug resistance (Yasodha et al. 2023; Qu et al. 2019). Recently, the role of DCLK1 in fostering immune suppression within the tumor microenvironment has garnered increasing attention. In breast cancer, DCLK1 increases in the stem cell-rich subtype of Triple-negative breast cancer (TNBC) and could promote immune escape through the IL6/STAT3 pathway (Liu, et al. 2023). In pancreatic cancer, inhibition of DCLK1 could down-regulate PD-L1 expression by regulating YAP in the Hippo pathway (Yan et al. 2020). Growing evidence indicates DCLK1 overexpression within the CCA cancer stem cells (CSCs) subpopulation and in serum samples from patients with CCA, and might be a potential predictive biomarker for early diagnose in CCA(Nevi et al. 2021; Meadows and Francis 2021; Andresen et al. 2015). However, it is still unexplained how DCLK1 impacts the progression of cholangiocarcinoma and the particular procedures by which it encourages tumor advancement in CCA.

In this study, we explore the physiological effect of DCLK1 in CCA proliferation, migration, invasion, and EMT processes. Mechanistically, our results revealed that DCLK1 promoted CCA progression and EMT process through the PI3K/AKT/mTOR pathway. Consequently, our research underscores the significance of elevated DCLK1 expression as a crucial marker indicative of an unfavorable prognosis in CCA. Moreover, individuals exhibiting heightened DCLK1 levels are poised to derive substantial benefits from therapeutic interventions targeting the PI3K/AKT/mTOR pathway, thereby presenting a promising avenue for improving patient prognosis.

## Materials and methods

### Cell lines and culture

The cholangiocarcinoma cell lines (RBE and HCCC 9810) and the human embryonic kidney cell line 293 T were acquired from the American Type Culture Collection (ATCC). RBE and HCCC 9810 were cultured in RPMI-1640 medium (Sigma, USA), while 293 T cells were cultured in DMEM medium (Sigma, USA). Both mediums were supplemented with 10% fetal bovine serum (FBS, Ausbian, Australia) and 1% penicillin/streptomycin (Solarbio, Beijing, China). Incubation of all cells took place in a humidified incubator at 37℃ with 5% CO_2_.

### Establishment of DCLK1 knockout and overexpression cell lines

For the construction of a DCLK1-knockout cell line, we employed the clustered regularly interspaced short palindromic repeats (CRISPR)/Cas9 technique. The sgRNA sequences utilized were listed as follows:

Oligo1-DCLK1- 5′-CACCGGAGTAGAGAGCTGACTACCA-3′

Oligo2-DCLK1- 5′-AAACTGGTAGTCAGCTCTCTACTCC-3′

These two sgRNA oligomers were cloned into the predigested lentiCRISPR V2 plasmid. Following transfection into 293 T cells alone with psPAX_2_ and pMD_2_G (packaging plasmids) using DNA transfection reagent (Neofect (Beijing) Biotech Co., Ltd), the lentivirus was harvested from the cell culture supernatant after 72 h and filtrated using a 0.45 μm filter. RBE cells underwent infection with lentivirus and polybrene, and subsequent stable transfection cells were screened using 2 μg/ml of puromycin.

To obtain a DCLK1-overexpressing cell line, the human DCLK1 overexpression lentivirus plasmid (pCDH-DCLK1-GFP) was purchased from Beijing AUGCT (China). The DCLK1-OE lentivirus transfection and infection procedures mirrored those described above. Ultimately, the target cells were screened using fluorescence microscopy.

### DCLK1 expression Rescue and inhibition in cell lines

To confirm the regulatory function of DCLK1 expression in CCA, sequence-modified DCLK1 was stably transfected using a linearized vector, thereby restoring DCLK1 expression in DCLK1-KO cell lines. Assess whether reinstating DCLK1 expression can counteract the tumor-suppressive effect induced by DCLK1 knockout. The verification of DCLK1 expression rescue was conducted through Western blot. Furthermore, we used two different DCLK1 inhibitors (LRRK2-IN-1 and DCLK1-IN-1) with a final concentration of 2.5 μM to inhibit the expression of DCLK1 in HCCC 9810 DCLK1-OE cell lines. The inhibition effect of these two compounds on DCLK1 expression was validated by Western blot.

### Transwell assays

To investigate the migratory and invasive capacities of cholangiocarcinoma cells, transwell assays were conducted using 8.0 μm pore membrane chambers placed in a 24-well plate. In order to perform the migration assay, we prepared cell suspensions using serum-free medium and filled the top chambers with 200μL cell suspensions (containing 2 × 10^4^ cells). In the invasion assay, Matrigel (BD Bioscience, USA), diluted at a 1:8 ratio with a serum-free media, was applied to the upper chambers before cell inoculation and allowed to incubate for 2 h at 37℃. In each chamber, 600μL of cell culture medium containing 10% FBS served as the chemoattractant at the bottom. Following a 24-h incubation period, the migrated and invaded cells were fixed for 10 min using 4% paraformaldehyde and then stained for 20–30 min with 0.1% crystal violet, and observed under the microscope. 4–5 fields/well were selected and analyzed with the Image J software.

### Real-time cellular analysis, RTCA

RTCA, a cellular biosensor, operates on the principle of electrical impedance and utilizes electronic cell sensor arrays. This innovative cell detection technology enables real-time monitoring of cell dynamics. It provides insights into changes in cell survival, growth, and other pertinent information. E-plates were utilized, with 5 × 10^3^ cells/well seeded and incubated in an RTCA detector at 37 ℃. The automatic cell proliferation recording of each group occurred at 15-min intervals.

### Colony formation assay

We planted 300 Cholangiocarcinoma cells into each well of a 6-well plate, and cells were cultured for 2-week intervals at 37 ℃ with 5% CO_2_. After using 4% paraformaldehyde, the colonies were fixed for half-hour, and stained for 10 min with 0.1% crystal violet. The dyed colonies were observed and recorded under the light microscope.

### Western blotting

Total cellular protein was extracted using RIPA lysis buffer (Beijing Solarbio Science and Technology Co., Ltd.), supplemented with 1% PMSF and 1% phosphatase inhibitor. After that, the BCA Protein Assay Reagent (Thermo Fischer, USA) was utilized to determine the concentrations of whole-cell protein. After boiling at 95 ℃ for 10 min, the fully denatured protein samples (50 μg) were loaded to the sample pores, separated using 10% SDS-PAGE gels, and subsequently transferred onto PVDF membranes (Millipore Corporation, USA). To block the membranes, 8% skim milk was applied for 1 h and incubated with primary antibodies at 4℃ overnight.

The primary antibodies used included: anti-DCLK1 was purchased from Abcam; anti-E-cad, anti-N-cad, anti-ZO-1, anti-Snail, anti-Slug, anti-Vimentin, anti-ZEB1 and anti-β-actin were obtained from Proteintech; anti-AKT, anti-Phospho-AKT, anti-PI3K, anti-Phospho-PI3K, anti-mTOR and anti-Phospho-mTOR were obtained from Cell Signaling Technology. Following three washes with 1 × TBST, membranes underwent a one-hour incubation with secondary antibodies and subsequent measurement using ECL reagents (E1080, LABLEAD). The specific information of antibodies, including dilution ratio, brand, and product numbers were attached to the supplement (Supplementary Table 1).

### Real-time PCR

Total RNA extraction employed TRIzol reagent (Invitrogen, USA), followed by reverse transcription into cDNA using Hifair^®^ III 1st Strand cDNA Synthesis SuperMix for qPCR (gDNA digester plus) (11141ES60, Yeasen, China). Real-time PCR was conducted using SYBR Green Premix (Invitrogen, USA) on the 7500 Sequence Detection System (Applied Biosystems, China). GAPDH served as a reference gene, and the relative mRNA expression of target genes was calculated using the 2^−ΔΔC(t)^ method. Supplementary Table 2 contains a list of specific primer sequences used in this article.

### Immunohistochemistry (IHC)

To assess the expression of DCLK1 in cholangiocarcinoma and normal tissues, a CCA tissue microarray (including 79 CCA tissue) was purchased from Bioaitech. In addition, samples of primary tumor tissue from 49 CCA patients were obtained from Beijing Chao-Yang Hospital. All tissues included in this research obtained ethical approval by the ethics committee of Beijing Chaoyang Hospital, Capital Medical University. These tissue sections were roasted at 65℃ for two hours and dewaxed by soaking in xylene solution. After gradient hydration, antigen retrieval, endogenous peroxidase elimination, and goat serum blocking, all tissue sections were incubated with anti-DCLK1 (1:500, Abcam, ab31704) at 4 ℃ overnight. The secondary antibody was incubated after the tissue sections were returned to room temperature. DAB chromogen and hematoxylin were used to stain the slices and to assess the expression levels of DCLK1. After dehydration and sealing, all slides were observed and recorded under a microscope, and the IHC score of the staining density and proportion of DCLK1-positive cells in each slide was calculated using Image J software. Using the IHC score, CCA patients were categorized into groups based on DCLK1 expression levels, distinguishing between those with high and low DCLK1 expression.

### Multiplex immunohistochemistry (mIHC) analysis

To intuitively demonstrate the correlation between DCLK1 expression and the epithelial mesenchymal transformation (EMT) process, multiplex immunohistochemistry (mIHC) analysis was performed on the tissue sections mentioned above, using the Opal™ 4-colour Manual IHC kit (PerkinElmer, USA). All slides were incubated with anti-DCLK1 (ab31704, 1:500) opal 520, anti-E-cadherin (20874-1-AP, 1:3000) opal 690, and anti-Vimentin (10366–1-AP, 1:3000) opal 570, and the nuclear stain was conducted with DAPI. Multiple stained sections were scanned by Beijing Bodu Hengyi Technology company under the same exposure time conditions, and demonstrated through NDP. View 2 software. To further explore the expression of each marker, the Image J software was used to separately assess the mean fluorescence intensity of each channel.

### Immunofluorescence (IF) staining

CAA cells were seeded on a cover slide inside a 24-well plate until the cell density reached 70%. After fixation with 90% ethanol, 0.5% Triton X-100 was used to break the cell membrane. Primary antibodies were added separately and covered each slide at 4 ℃ overnight: anti-DCLK1 (ab31704, 1:150), anti-E-cadherin (20874–1-AP, 1:150) and anti-Vimentin (10366–1-AP, 1:150). The next day, Rhodamine-Conjugated Anti-Rabbit IgG (1:150) was incubated for one hour. All cover slides were sealed with a capping agent containing DAPI dye and observed under the fluorescence microscope.

### Subcutaneous graft tumor model in nude mice

Female BALB/c nude mice (5–6 weeks old), acquired from Charles River Laboratory (Beijing, China), were randomly allocated into two groups (6 mice per group) for the establishment of the Subcutaneous tumor model. Each mouse received a subcutaneous injection of 100μL RBE and RBE DCLK1-KO cells (containing 2 × 10^6^ cells). The tumor sizes were measured every 4–5 days and calculated via the formula:Volume = (Length × Width^2^)/2. Subcutaneous lumps were obtained and paraffin was embedded and fixed for mIHC analysis. All animal experiments conducted in this research received approval from the Institutional Animal Care and Use committee of Capital Medical University.

### The cancer genome atlas (TCGA) data and RNA sequencing

The level 3 mRNA expression data of DCLK1 (RNAseq (polyA + IlluminaHiSeq)) of 45 CCA patients in the TCGA database (Cohort: TCGA Bile Duct Cancer (CHOL)) were collected from UCSC Xena (http://xena.ucsc.edu/) in order to examine the differences in DCLK1 expression between the tumor and nearby non-tumor tissues. In addition, using the R package “DESeq2”, RNA-seq analysis was carried out to determine the differentially expressed genes (DEGs) across CCA cell lines: RBE vs. RBE DCLK1-KO, HCCC 9810 vs. HCCC 9810 DCLK1-OE. The principles for DEG screening were as follows: False Discovery Rate (FDR) < 0.05 and fold change of ≥ 2, or ≤ 2. The gene functions of DEGs were revealed by Gene ontology (GO) analysis, and the “clusterProfiler” R package was used to target the DEGs enrichment pathway in Kyoto Encyclopedia of Genes and Genomes (KEGG) study.

### Statistical analyses

GraphPad Prism 9.0 was used to analyze the experimental data. Continuous variables, including gene expression levels, clone counts, and the number of invaded and migrated cells, were all presented as mean ± SD. Comparison between groups were conducted using the independent *t* test. The outcome for CCA patients was defined as overall survival (OS), with survival analyses conducted using Kaplan–Meier curve and evaluated with the log-rank test. Bioinformatics analysis and statistical mapping of RNA sequencing results for cell lines, including DEGs analysis, GO and KEGG analysis, were performed using R software (Version 4.1.3). Statistical significance was defined as *p < 0.05, **p < 0.01, ***p < 0.001, ****p < 0.0001.

## Result

### Enhanced DCLK1 expression in cholangiocarcinoma correlates with poor prognosis

**Fig. 1:**
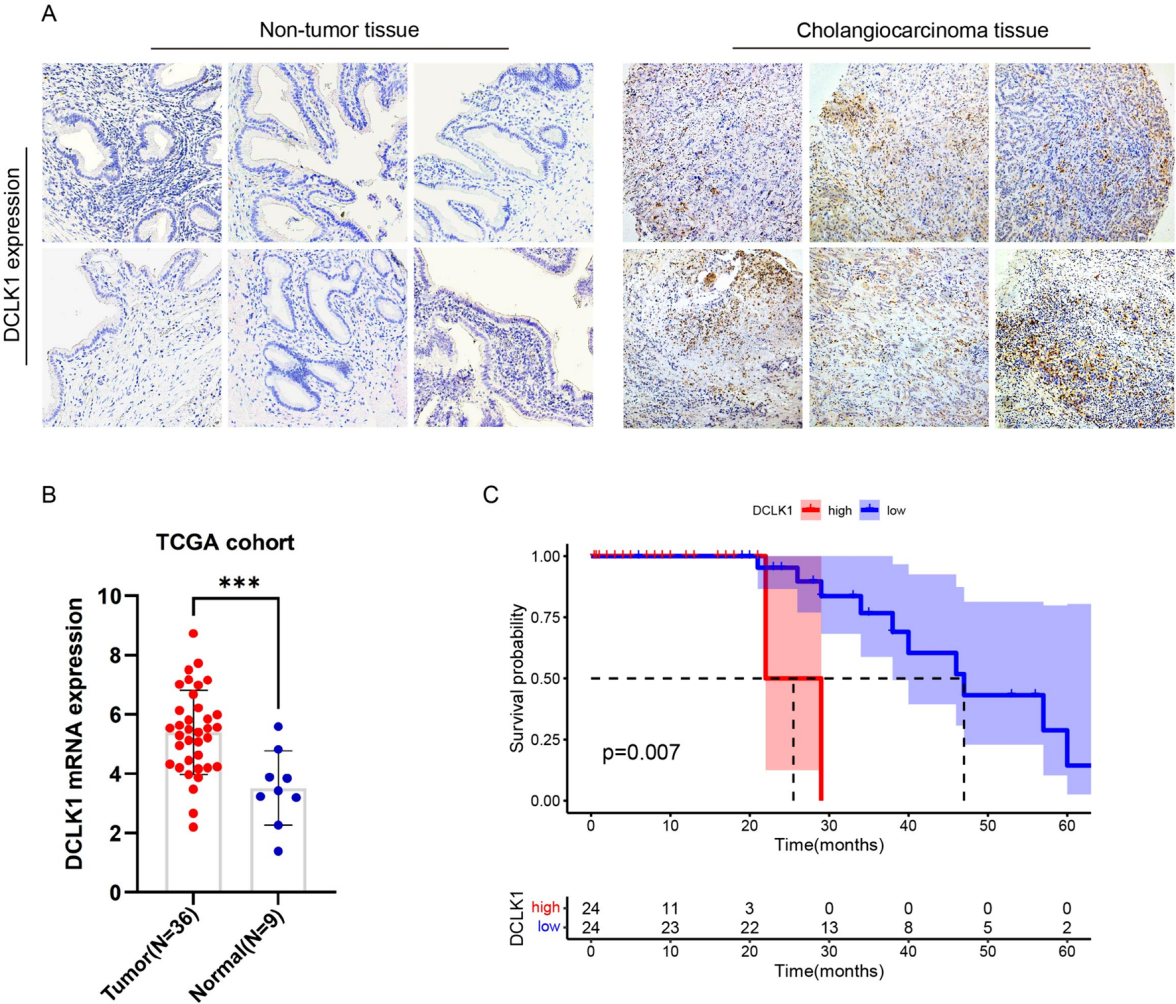
DCLK1 is up-regulated in Cholangiocarcinoma and associated with poor prognosis. **A** Immunohistochemical staining of the DCLK1 expression in CCA tissues (n = 79) compared to non-tumor bile duct tissues (n = 10); **B** TCGA analysis of the mRNA expression of DCLK1 in CCA (n = 36) compared to para-cancer tissues (n = 9), P < 0.001; **C** High expression of DCLK1 is associated with poor outcomes in CCA patients, P = 0.007

To assess DCLK1 expression in both tumor and non-tumor tissue, IHC detection was conducted on a cholangiocarcinoma tissue microarray (containing 79 tumors) and surgical tissues from cholecystitis patients (n = 10). As depicted in Fig. 1A, DCLK1 expression exhibited a notable upregulation in CCA samples compared to non-tumor tissues. Additionally, our findings from the TCGA-CCA cohort indicated a significant elevation in DCLK1 mRNA expression in CCA tissue (5.39 ± 1.40) compared to para-cancer tissue (3.52 ± 1.18) (t = 3.63, P < 0.001, Fig. 1 B), aligning with the results observed in our IHC analysis. In order to further examine the potential correlation between DCLK1 expression levels and patient survival, we examined a cohort comprising 49 CCA patients from Beijing Chao-Yang Hospital, with survival follow-ups. The specific clinical and tumor pathology information was provided in Supplementary Table 3. Paraffin sections from tumor tissues were acquired, and DCLK1 expression was assessed through IHC. Image J software was utilized to calculate the IHC score for each slide, defined as the number of positive cells in a 40 × field of view. Using the median IHC score (208.5) of all paraffin sections, we categorized the 49 CCA patients into two groups: high and low DCLK1 expression. The survival analysis, conducted using the Kaplan–Meier (K–M) curve, revealed that patients with elevated DCLK1 expression experienced a reduced overall survival (OS) compared to those with lower levels of DCLK1 expression (P = 0.007, Fig. 1C). These findings underscore the association between DCLK1 expression and cholangiocarcinoma progression, indicating that higher expression levels correlate with a poorer prognosis.

### DCLK1 promotes the proliferation, invasion, migration, and EMT process of CCA cells

**Fig. 2:**
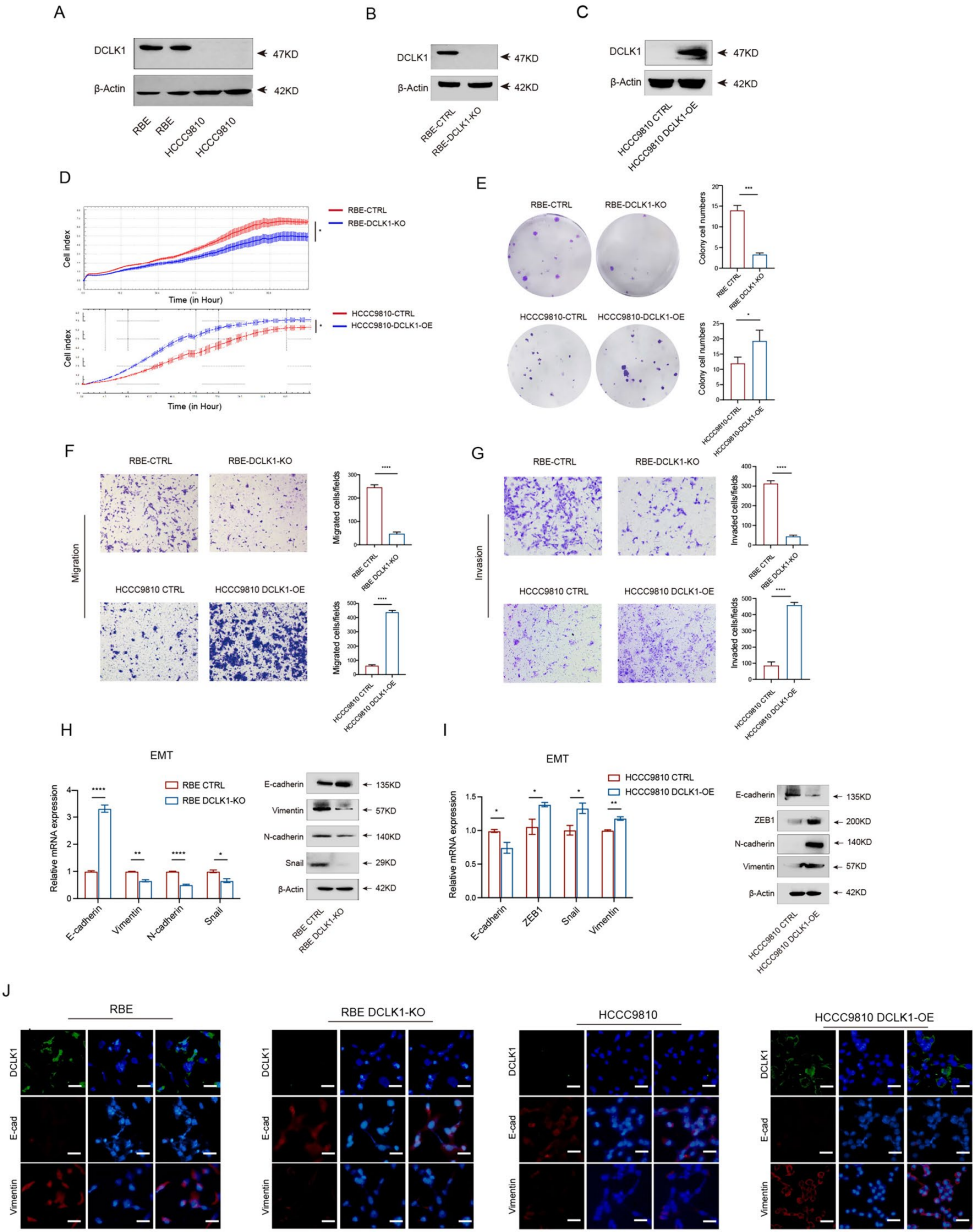
DCLK1 plays a role in promoting progression and EMT activation in CCA cells. **A** The basal protein expression of DCLK1 in two CCA cell lines; **B** and **C**. knockout of DCLK1 in RBE and overexpression of DCLK1 in HCCC9810; **D** and **E** RTCA result and colony-formation assay showing that DCLK1 supports tumor proliferation; **F** and **G** Transwell assay showing that DCLK1 promotes CCA cell migration and invasion; **H** and **I** RT-PCR and Western blot analysis of the EMT-related markers performing that DCLK1 results in the activation of the EMT procedure. **J** The relationship between DCLK1 and EMT-related markers detection by Immunofluorescence staining

To delve deeper into the distinct biological role of DCLK1 in driving CCA progression, a series of in vitro experiments were conducted. Initially, we assessed the baseline expression of DCLK1 in two cholangiocarcinoma cell lines, RBE and HCCC9810. Western blot results revealed a significantly higher expression level of DCLK1 in RBE compared to HCCC9810 (Fig. 2A). Subsequently, DCLK1 was knocked out in RBE through the CRISPR/Cas9 technique, while DCLK1 was overexpressed in HCCC9810 via lentivirus transfection. Successful establishment of stable RBE DCLK1-KO and HCCC9810 DCLK1-OE cell lines was confirmed using Western blot analysis (Fig. 2B, C). RTCA results showed that depletion of DCLK1 expression inhibited the proliferation of RBE cells. The cell proliferation index of RBE was 6.75 ± 0.24, compared to 4.98 ± 0.45 in RBE DCLK1-KO (t = 5.10, P = 0.015, Fig. 2D upper panel). In contrast, overexpression of DCLK1 enhanced the proliferation of HCCC9810 cells. The cell proliferation index of HCCC9810 was 6.28 ± 0.19, compared to 7.13 ± 0.23 in HCCC9810 DCLK1-OE (t = 3.69, P = 0.034, Fig. 2D lower panel). The same conclusion was also confirmed in colony formation assays, where cell lines with higher expression of DCLK1 (RBE and HCCC9810) performed more monoclonality. The clone counts of RBE was 14.00 ± 1.63, compared to 3.33 ± 0.47 in RBE DCLK1-KO (t = 8.88, P < 0.001). The clone counts of HCCC9810 was 12.00 ± 1.63, compared to 19.33 ± 2.87 in HCCC9810 DCLK1-OE (t = 3.14, P = 0.035, Fig. 2E). Moreover, transwell assays were employed to examine the impact of DCLK1 expression on the migration and invasion capacity of CCA cells. We found that the number of migrated cells with DCLK1 overexpressing HCCC9810 group was 431.67 ± 6.13, which was significantly higher than that of HCCC9810 control cells (66.67 ± 1.25, t = 82.54, P < 0.0001). Similarly, the migration number of RBE DCLK1-KO cells was 47.00 ± 3.74, which was significantly lower than that of the control group (255.00 ± 8.83, t = 30.67, P < 0.0001). The same trend was found in the invasion experiment. These outcomes indicated that overexpression of DCLK1 enhanced both the migration and invasion, and conversely, knockout of DCLK1 decreased both (Fig. 2F, G).

Prior studies have highlighted DCLK1's impact in propelling the epithelial-mesenchymal transition (EMT) across a spectrum of malignancies. Nonetheless, the extent of its regulatory influence in cholangiocarcinoma is yet to be fully understood. To explore this aspect, we performed RT-PCR and Western blot of EMT-related genes in RBE vs. RBE DCLK1-KO and HCCC9810 vs. HCCC9810 DCLK1-OE. The results show that in RBE DCLK1-KO cells, mesenchymal markers including Vimentin, N-cadherin, and Snail were significantly down-regulated, while the epithelial marker (E-cadherin) was up-regulated (Fig. 2H). Conversely, DCLK1 overexpression was linked to EMT activation by inhibiting E-cadherin expression and enhancing ZEB1, N-cadherin, and Vimentin (Fig. 2I). Immunofluorescence (IF) staining of cells on glass slides further illustrated the negative correlation of DCLK1 expression with E-cadherin and its positive correlation with Vimentin (Fig. 2J). These findings underscore the pivotal role of DCLK1 in the EMT process, establishing it as a significant risk factor for the progression of CCA.

### DCLK1 knockout inhibits the formation of subcutaneously transplanted tumor in mice

**Fig. 3:**
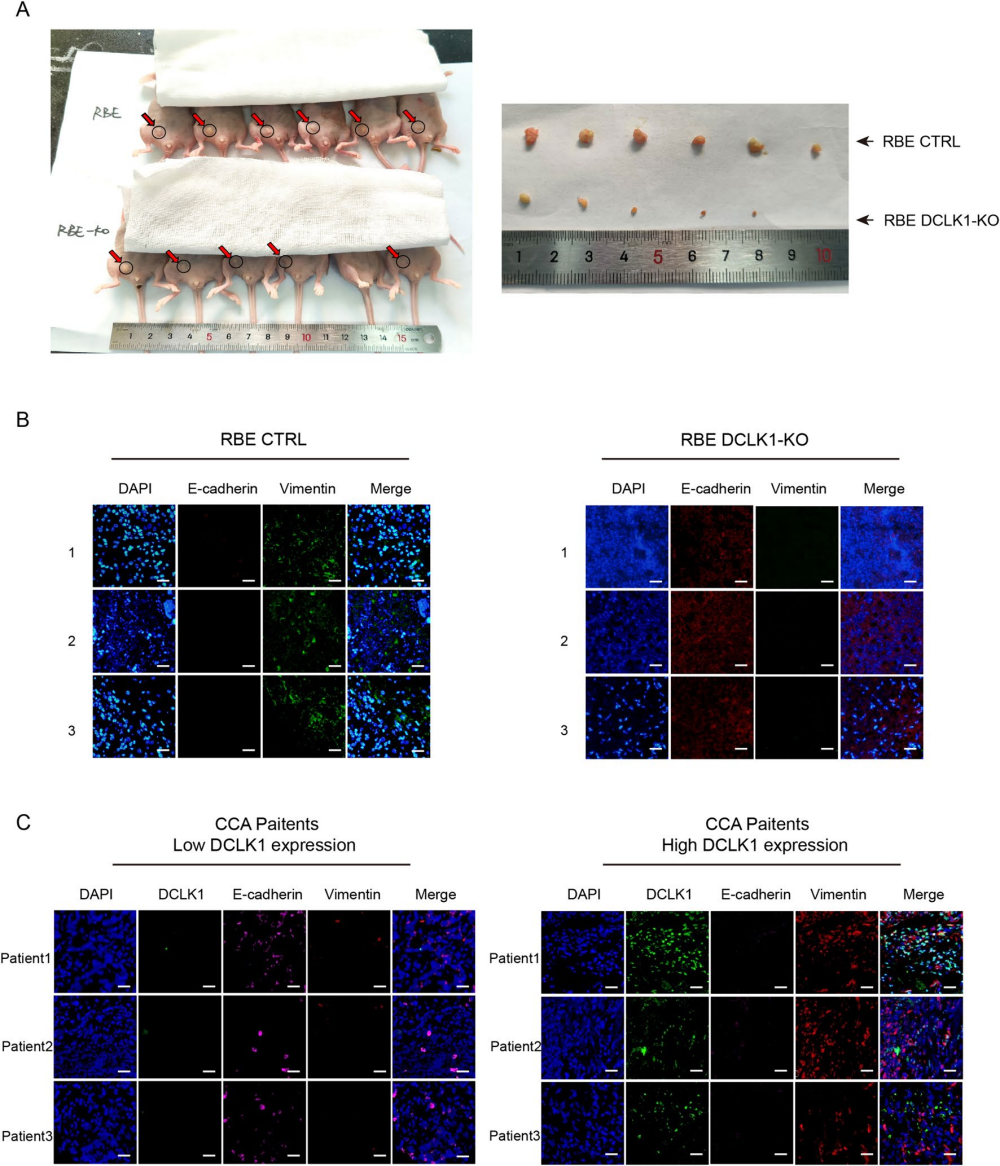
DCLK1 promotes tumor proliferation and EMT activation. **A** Knockout of DCLK1 inhibits the tumor growth of mice; **B** mIHC staining results of E-cadherin and Vimentin in subcutaneous neoplasia of mice indicate that knockout of DCLK1 inhibits EMT activation; **C** mIHC results of 49 human CCA tissues indicate that patients with high expression of DCLK1 are accompanied by high expression of Vimentin and low expression of E-cadherin

Subsequently, to further investigate the function of DCLK1 in augmenting tumor growth in vivo, we employed BALB/c nude mice to establish a subcutaneous transplant tumor model. Cells from both RBE CTRL and RBE DCLK1-KO groups, in the logarithmic growth phase, were subcutaneously injected into the abdomen of mice (6 mice per group). Notably, the RBE CTRL group displayed a significantly larger tumor size compared to the RBE DCLK1-KO group, indicating a substantial impairment in subcutaneous tumor development upon DCLK1 deactivation (Fig. 3A). Then, we obtained the subcutaneous tumor tissues for mIHC about the expression of E-cadherin, and Vimentin. The results demonstrated that tumor tissues from the RBE CTRL group exhibited lower E-cadherin expression and higher Vimentin expression compared with the RBE DCLK1-KO group (Fig. 3B). Therefore, we provided highly convincing evidence that DCLK1 promote the progression of CCA cells in vivo.

### DCLK1 regulates the EMT process in CCA patients

In addition, we performed mIHC in samples from 49 CCA patients to assess the protein expression of DCLK1, E-cadherin, and Vimentin. Based on the median DCLK1 positive cell numbers (median IHC score = 208.5), patients were categorized into two groups: the DCLK1 high expression group and the DCLK1 low expression group. It was observed that individuals with elevated DCLK1 expression also exhibited heightened levels of Vimentin and reduced expression of E-cadherin (Fig. 3C). These outcomes provided compelling evidence supporting the role of DCLK1 in triggering the EMT process.

### DCLK1 activates the PI3K/AKT/mTOR pathway

**Fig. 4:**
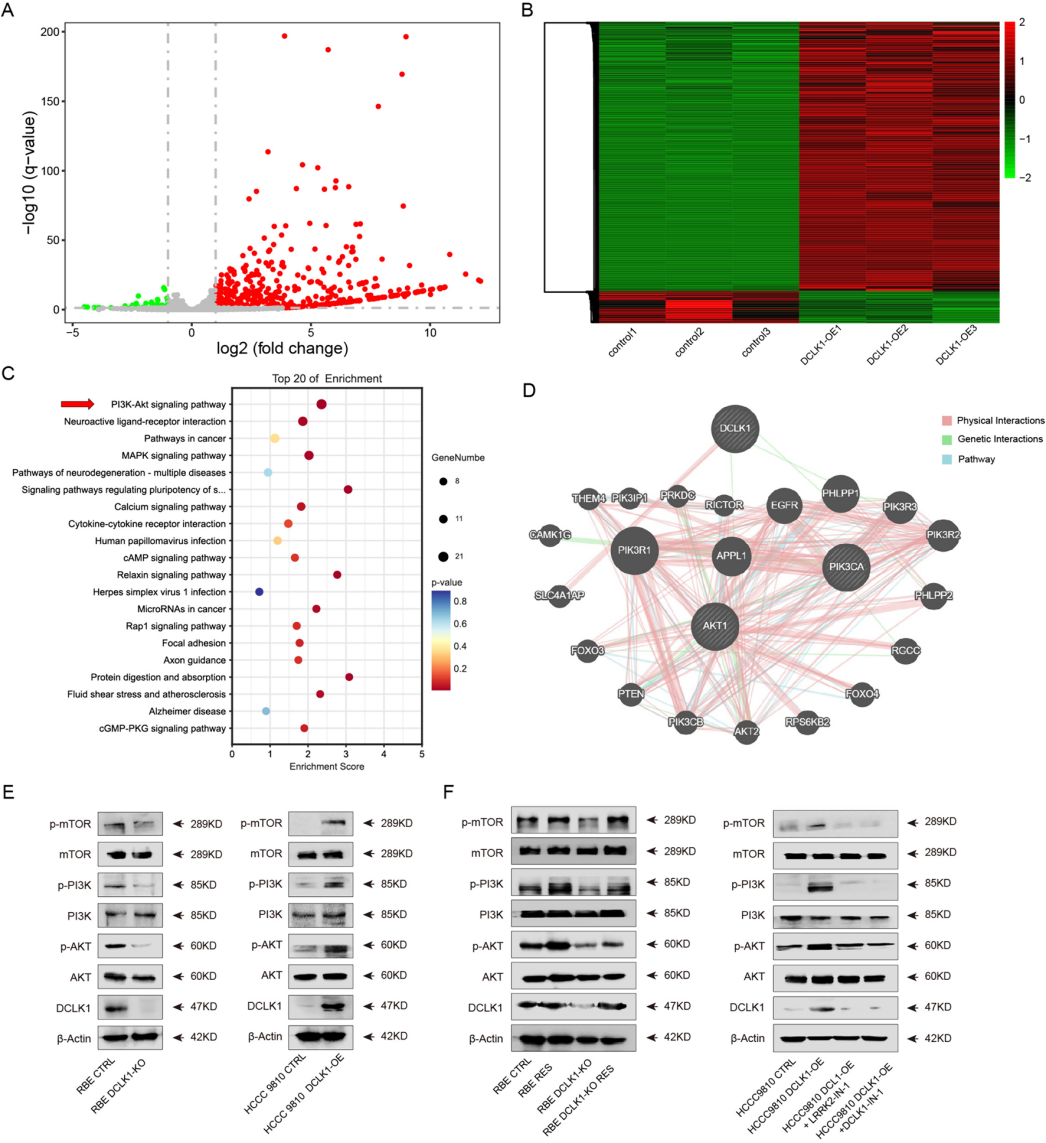
DCLK1 activates PI3K/AKT/mTOR pathway. **A** Volcano map of Different expressed genes (DEGs) between HCCC9810 and HCCC9810 DCLK1-OE; **B** Heatmap analysis of HCCC 9810 and HCCC 9810 DCLK1-OE; **C** KEGG analysis of DEGs screens the PI3K/AKT pathway as the key pathway related with DCLK1; **D** The GeneMANIA network shows a correlation between DCLK1 and PI3K/AKT/mTOR signaling pathway based on the physical interactions, pathway, and genetic interactions; **E** and **F** Western blots results of DCLK1 mediated the activation of the PI3K/AKT pathway

To unravel the specific biological mechanism through which DCLK1 promotes CCA progression, RNA transcriptome sequencing was conducted on HCCC9810, and HCCC9810 DCLK1-OE cells. The comparative analysis between HCCC9810 and HCCC9810 DCLK1-OE revealed 493 DEGs in HCCC9810 DCLK1-OE cells, including 38 down-regulated genes and 455 up-regulated genes (Fig. 4A and B). Notably, KEGG analysis highlighted the predominant enrichment of DEGs in the PI3K/AKT pathway (Fig. 4C). Correspondingly, we delved further into the relationship between DCLK1 and the PI3K/AKT/mTOR pathway utilizing the GeneMANIA database. The gene network apparently indicated that DCLK1 and PI3K/AKT/mTOR signaling pathway had network relationships based on the physical interactions, pathway, and genetic interactions (Fig. 4D). Therefore, based on the results of RNA-Seq analysis, we further explored whether DCLK1 promotes the development of cholangiocarcinoma by activating the PI3K/AKT/mTOR signaling pathway.

Firstly, we conducted a Western blot to investigate the expression of PI3K/AKT/mTOR pathway-related markers in CCA cells. We found that the knockout of DCLK1 significantly reduced the protein levels of phosphor-mTOR, phosphor-PI3K, and phosphor-AKT. In contrast, overexpression of DCLK1 stimulated the PI3K/AKT/mTOR pathway by upregulating p-mTOR, p-PI3K, and p-AKT (Fig. 4E). In this way, we proposed that DCLK1 was strongly correlated with the activation of the PI3K/AKT/mTOR pathway. Subsequently, DCLK1 expression was rescued in RBE DCLK1-KO cells and then we extracted the total cell protein of RBE-DCLK1-OE and RBE DCLK1-KO-RES for Western blot to verify the expression of DCLK1 and other markers of PI3K/AKT/mTOR pathway. The results revealed that DCLK1 expression was successfully rescued in RBE DCLK1-KO cells. Besides, rescuing of DCLK1 expression could re-activate the PI3K/AKT pathway, including the upregulation of p-mTOR, p-PI3K, and p-AKT (Fig. 4 left panel). Subsequently, we used two distinct DCLK1 inhibitors, LRRK-2 (2.5 μM) and DCLK1-IN-1 (2.5 μM), to suppress DCLK1 expression in HCCC9810 DCLK1-OE cells. The activation of the PI3K/AKT/mTOR pathway in HCCC 9810 DCLK1-OE cells returned to baseline following treatment with DCLK1 inhibitors (Fig. 4F right panel). These findings indicate that elevated levels of DCLK1 in cholangiocarcinoma markedly stimulate the PI3K/AKT/mTOR signaling pathway.

### DCLK1 promotes CCA progression and EMT process via the PI3K/AKT/mTOR pathway

**Fig. 5:**
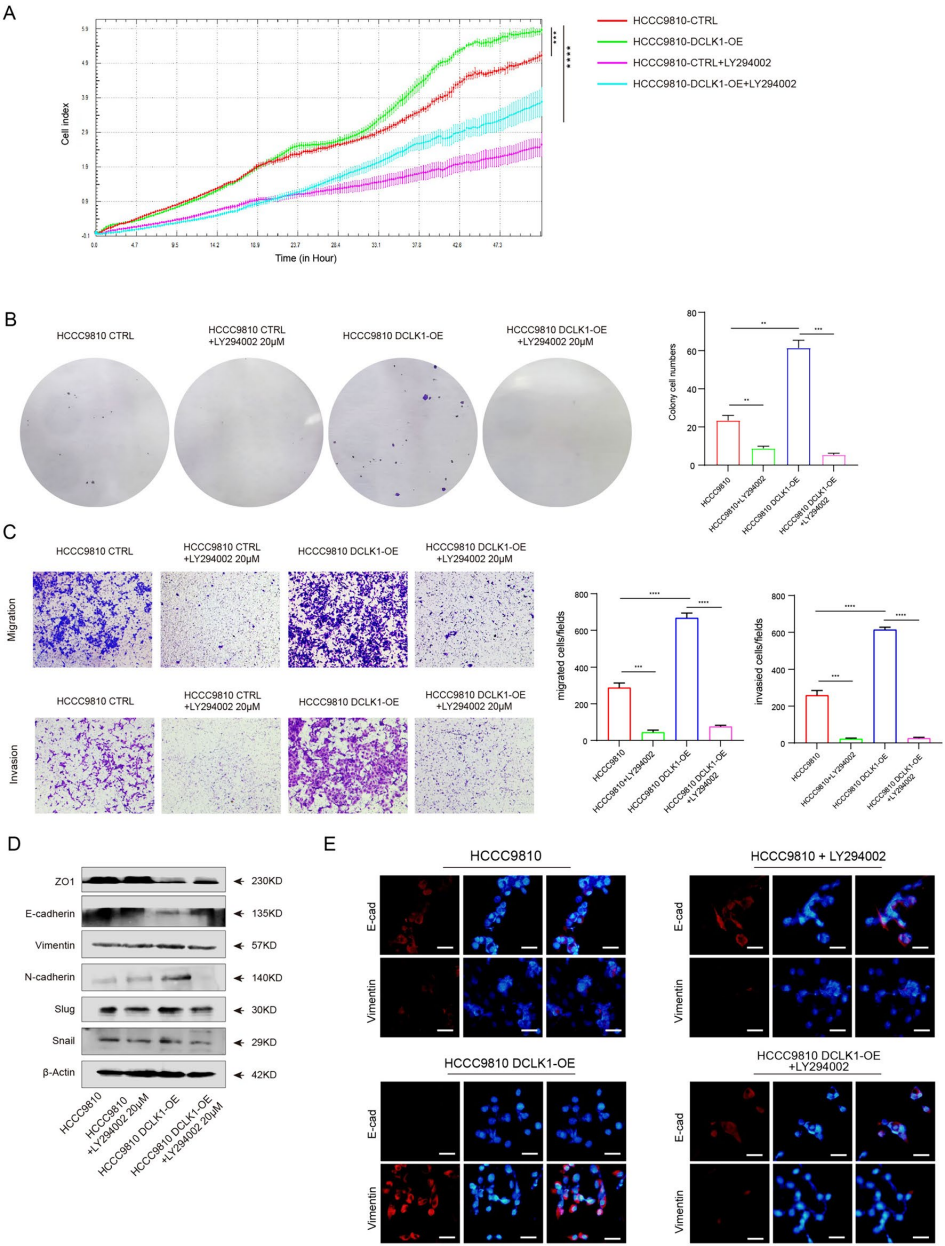
DCLK1 promotes the progression in CCA through the activation of the PI3K/AKT/mTOR pathway. **A** and **B** RTCA analysis and colony-formation assay showing a reduction of DCLK1 mediated proliferation after the treatment of the PI3K/AKT pathway inhibitor LY294002; **C** Treating with LY294002 inhibits the DCLK1 mediated migration and invasion; **D** and **E** Western blot and IF staining results of the EMT status after LY294002 treatment in DCLK1 overexpression cells

To further verify whether DCLK1 promotes the malignant phenotype of CCA through stimulating the PI3K/AKT/mTOR pathway, we employed LY294002, a specific inhibitor of the PI3K/AKT/mTOR pathway, to evaluate the special impact of DCLK1 on CCA progression regulation. RTCA was used to record the proliferation curves of HCCC9810, HCCC9810 DCLK1-OE, HCCC9810 + LY294002, and HCCC9810 DCLK1-OE + LY294002. The outcomes revealed a significantly higher proliferation capacity in DCLK1-OE cells (5.80 ± 0.07) compared to control cells (5.06 ± 0.10, t = 9.86, P < 0.001). However, treatment with LY294002 (20 μM) led to a sharp decrease in the proliferation index in both DCLK1 overexpression cells (3.85 ± 0.34, t = 9.69, P < 0.0001) and control cells (2.35 ± 0.40) (Fig. 5 A). The clone counts of DCLK1 overexpressing HCCC9810 group was 61.33 ± 5.79, which was significantly higher than that of HCCC9810 control cells (23.33 ± 3.86, t = 7.72, P = 0.002) and DCLK1 overexpressing cells treated with LY294002 (5.33 ± 1.25, t = 13.37, P < 0.001).The colony formation assay also confirmed that the PI3K/AKT/mTOR pathway inhibitor could significantly reduce the proliferation of CCA cells mediated by high expression of DCLK1 (Fig. 5B). Moreover, transwell experiments were performed to compare the invasion and migration ability of each group, including HCCC9810, HCCC9810 DCLK1-OE, HCCC9810 + LY294002 and HCCC9810 DCLK1-OE + LY294002. The results demonstrated that the number of invaded and migrated cells in the DCLK1-overexpressed group was significantly higher than that in the control group and the LY294002 treatment group (P < 0.0001, Fig. 5C). Apparently, the PI3K/AKT/mTOR pathway inhibitor attenuated the promotion of invasion and migration by high DCLK1 expression in CCA cells. Additionally, protein levels of EMT-related markers in these four groups were assessed through Western blot and IF. As depicted in Fig. 5D, E, inhibition of the PI3K/AKT/mTOR pathway reversed the EMT process, manifesting as increased expression of E-cadherin and ZO1, and decreased expression of N-cadherin, Vimentin, Snail, and Slug. Together, these findings suggested that DCLK1 promotes CCA progression via the PI3K/AKT/mTOR pathway.

## Discussion

Cholangiocarcinoma originates in bile duct epithelial cells and is highly malignant with a poor prognosis. The highest incidence and mortality rates of CCA have been reported in Eastern Asia, especially in China and Thailand due to chronic infections including liver fluke infections and Hepatitis B and C viruses (Rizvi et al. 2017; Labib et al. 2019). Currently, hepatic resection remains the only curable treatment for patients with early-diagnosed CCA. However, more than half of CCA patients are unresectable and can only receive systemic treatment to prolong survival (Mazzaferro et al. 2020). Despite being the primary treatment regimen for advanced CCA patients, the combination of Cisplatin and Gemcitabine has not yielded enhanced survival outcomes, with the median overall survival for individuals with CCA hovering around a mere 11 months (Valle, et al. 2010; Okusaka et al. 2010). Recently, with the deepening cognition and development of immunotherapy, the treatment of CCA has ushered in a new era. The TOPAZ-1 trial revealed that combined durvalumab significantly prolonged overall compared with chemotherapy alone (median OS 12.8 months vs. 11.5 months, HR = 0.80, 95%CI = 0.66–0.97, P = 0.021) (Oh, et al. 2022). Accordingly, the Food and Drug Administration (FDA) has approved durvalumab in combination with chemotherapy (Gemcitabine + Cisplatin) for the first-line treatment of patients with advanced biliary tract cancer. Nevertheless, the five-year survival rate for CCA patients remains 10%. Considerable progress is still needed to enhance the prognosis for patients with CCA (Sahu et al. 2022; Greten et al. 2023). Therefore, our research focused on this clinical dilemma, elucidating the precise molecular markers and key mechanisms that drive CCA progression.

DCLK1 has been identified as a specific marker of CSC in various gastrointestinal cancers including colon cancer, pancreatic cancer, and esophageal cancer. Substantial evidence revealed its roles in regulating tumor progression, metastasis, and EMT process (Cao et al. 2020; Ikezono et al. 2017; Dogra et al. 2023). Growing evidence has demonstrated that CCA is enriched with abundant cancer stem cells (CSCs). Consequently, the relationship between DCLK1 and CCA has drawn significant attention. Nevi et al. investigated DCLK1 overexpression in CSC-CCA populations and found that it plays an important role in tumor proliferation and viability (Nevi et al. 2021). By analyzing the promoter methylation status of candidate genes in CCA cell lines, Andresen et al. identified DCLK1 as a novel epigenetic biomarker for CCA, demonstrating high sensitivity and specificity (Andresen et al. 2012). These findings were further validated in biliary brush samples, indicating that DCLK1 detection may contribute to the early diagnosis of cholangiocarcinoma (Andresen et al. 2015). Although these studies have confirmed that DCLK1 is highly expressed in CCA and may serve as a potential biomarker for early diagnosis, there is no evidence elucidating the specific mechanism by which DCLK1 regulates the malignant biological behavior of CCA. Nevertheless, our study addresses this gap. We have not only confirmed that DCLK1 is overexpressed in CCA and promotes its proliferation, invasion, migration and EMT progression through a series of in vivo and vitro experiments, but also investigated its specific regulatory mechanisms.

In this project, we investigated whether DCLK1 contributes to CCA progression and elucidated the precise mechanisms. Firstly, our IHC results demonstrated that DCLK1 was significantly upregulated in human CCA tissues, but barely detected in non-tumor controls. For survival analysis, we observed that the upregulation of DCLK1 correlated with unfavorable outcomes. Next, we utilized DCLK1 knockout and over-expressed in two types of CCA cell lines to illustrate the regulatory effect of DCLK1 on CCA progression. Our results revealed that the overexpression of DCLK1 supported tumor proliferation and clonal formation, and promoted the migration and invasion capabilities of CCA cells. On the contrary, the elimination of DCLK1 expression reversed the malignant biological behaviors of CCA. In addition, we demonstrated that the overexpression of DCLK1 was accompanied by downregulation of E-cadherin and ZO1 (epithelial markers) and upregulation of N-cadherin, ZEB1, Snail, Vimentin, and Slug (mesenchymal markers), which presented the activation of EMT program. These findings suggested that DCLK1 may activate the EMT procedure in CCA. To further explore the underlying biological mechanism, we performed RNA transcriptome sequencing analysis and our results suggested that the PI3K/AKT/mTOR pathway is the key downstream pathway associated with DCLK1. The elevation of DCLK1 expression stimulated the PI3K/AKT/mTOR signaling pathway. The inhibition of the PI3K/AKT/mTOR pathway reversed DCLK1-mediated cell proliferation, migration, invasion, and the activation of the EMT program. Therefore, our findings parsed out that the regulatory effect of DCLK1 on CCA progression relies on the activation of the PI3K/AKT/mTOR pathway.

The human DCLK1 gene has been distinguished into two distinct subtypes, including the long (L)-isoform (DCLK1-isoform1, DCLK1-isoform2, ∼ 82KDa) and the short (S)-isoform (DCLK1-isoform3, DCLK1-isoform4, ∼ 47KDa), based on the origination of two different promoters, a 5'(α) promoter and a 3'(β) promoter, respectively (Kalantari, et al. 2022; Singh et al. 2016; Omori et al. 1998). These two subtypes of DCLK1 play different physiological and pathological functions in different cancers. Previous studies by our team have uncovered the irreplaceable role of DCLK1-S in the development of esophageal squamous cell carcinoma by stimulating the MAPK/ERK/MMP2 pathway to promote the EMT process (Ge et al. 2021). In this study, we also found that DCLK1-L (82KDa) was rarely expressed in the two CCA cell lines (data not shown), which indicated that DCLK1-S may be more significant in promoting malignant biological behavior and survival prediction in CCA.

Our results indicated that the PI3K/AKT/mTOR signaling pathway inhibitor could reverse DCLK1-mediated tumor progression, suggesting that CCA populations with high DCLK1 expression may benefit from therapies targeting the PI3K/AKT/mTOR pathway. In this way, it will be worth investigating DCLK1 as an auxiliary tool for identifying high-risk patients in future CCA screening. Overactivation of the PI3K/AKT/mTOR pathway is well known to be associated with the maintenance of stem cell characteristics, proliferation, differentiation, EMT program, and migration (Glaviano, et al. 2023; He, et al. 2021). In the last few decades, various clinical trials have shown prospective anti-tumor therapies targeting the PI3K/AKT/mTOR pathway. The PI3K inhibitor Alpelisib and the mTOR inhibitor Everolimus have been approved for new strategies in breast cancer (André et al. 2019; Piccart et al. 2014). In addition, the AKT inhibitor Ipatasertib has achieved milestone progression and a promising safety profile in the treatment of prostate cancer (Sweeney et al. 2021). Targeting the PI3K/AKT/mTOR pathway may be a new strategy for the treatment of CCA. Combined with our findings, we recommend the use of the PI3K/AKT/mTOR pathway inhibitors in CCA populations with high DCLK1 expression for better therapeutic outcomes. To provide further conclusive evidence in vivo experiments, we will further demonstrate the therapeutic effect of the PI3K/AKT/mTOR signaling pathway inhibitors on CCA with high DCLK1 expression by constructing animal models.

In conclusion, we have investigated that DCLK1 is up-regulated in CCA patients and results in poor outcomes. DCLK1 promotes CCA progression and the EMT process by stimulating the PI3K/AKT/mTOR pathway. DCLK1 may be a predictive biomarker of CCA prognosis, and targeting the PI3K/AKT/mTOR pathway may be a potential strategy to improve prognosis in CCA patients with higher DCLK1 expression.

### Supplementary Information

Below is the link to the electronic supplementary material.

Supplementary file1 (DOCX 193 KB)

Supplementary Figure 1. DCLK1 activates PI3K/AKT/mTOR pathway in RBE. A. Volcano map of Different expressed genes (DEGs) between RBE control and RBE DCLK1-KO; B. Heatmap analysis of RBE control and RBE DCLK1-KO; C. KEGG analysis of DEGs screens the PI3K/AKT pathway as the key pathway related with DCLK1 (JPG 307 KB)

## Data Availability

Data is available and can be obtained by contacting the corresponding authors (zdlzdl52973@sina.com and agybjcy@163.com).

## Abbreviations

DCLK1: Doublecortin-like kinase 1
CCA: Cholangiocarcinoma
CRISPR/Cas9: Clustered regularly interspaced short palindromic repeats/Cas9
IHC: Immunohistochemistry
mIHC: Multiplex immunohistochemistry
EMT: Epithelial mesenchymal transformation
KO: Knockout
OE: Overexpression
TCGA: The cancer genome atlas
DEGs: Differentially expressed genes
GO: Gene ontology
KEGG: Kyoto encyclopedia of genes and genomes
