# Supplementary material for: Targeting the PI3K/AKT/mTOR pathway offer a promising therapeutic strategy for cholangiocarcinoma patients with high doublecortin-like kinase 1 expression
Source: J Cancer Res Clin Oncol. 2024 Jul 9;150(7):342. doi: 10.1007/s00432-024-05875-3 (PMC11233391; doi:10.1007/s00432-024-05875-3)
Supplement: Supplementary file 1 — Supplementary file1 (DOCX 193 KB) [file 432_2024_5875_MOESM1_ESM.docx]

**Supplementary Table 1.** Detailed information of antibodies used for Western blot analysis.

|  | **Concentration** | **Company** |
| --- | --- | --- |
| **Primary antibodies (catalog number)** |  |  |
| Rabbit anti-DCLK1 (ab31704) | 1:1000 | Abcam |
| Rabbit anti-AKT (C67E7) | 1:1000 | Cell Signaling Technology |
| Rabbit anti-Phospho-AKT (D25E6) | 1:1000 | Cell Signaling Technology |
| Rabbit anti-PI3K (19H8) | 1:1000 | Cell Signaling Technology |
| Rabbit anti-Phospho-PI3K (E3U1H) | 1:1000 | Cell Signaling Technology |
| Rabbit anti-mTOR (7C10) | 1:1000 | Cell Signaling Technology |
| Rabbit anti-Phospho-mTOR (D9C2) | 1:1000 | Cell Signaling Technology |
| Rabbit anti-E-cadherin (20874-1-AP) | 1:1000 | Proteintech |
| Rabbit anti-N-cadherin (22018-1-AP) | 1:1000 | Proteintech |
| Rabbit anti-ZO-1 (21773-1-AP) | 1:1000 | Proteintech |
| Rabbit anti-Snail (13099-1-AP) | 1:1000 | Proteintech |
| Rabbit anti-Slug (12129-1-AP) | 1:1000 | Proteintech |
| Rabbit anti-Vimentin (10366-1-AP) | 1:1000 | Proteintech |
| Rabbit anti-ZEB1 (21544-1-AP) | 1:1000 | Proteintech |
| Rabbit anti-β-Acitin (20536-1-AP) | 1:1000 | Proteintech |
| **Secondary antibody (catalog number)** |  |  |
| HRP-conjugated Affinipure Goat Anti-Rabbit IgG(H+L) (SA00001-2) | 1:10000 | Proteintech |

**Supplementary Table 2.** Detailed information of primers used for RT-PCR.

| **Genes** | **Primers** | **Sequence (5’-3’)** |
| --- | --- | --- |
| E-cadherin | PCR-F | GTCACTGACACCAACGATAATCCT |
|  | PCR-R | TTTCAGTGTGGTGATTACGACGTTA |
| N-cadherin | PCR-F | TCAGGCGTCTGTAGAGGCTT |
|  | PCR-R | ATGCACATCCTTCGATAAGACTG |
| Vimentin | PCR-F | CCTGAACCTGAGGGAAACTAA |
|  | PCR-R | GCAGAAAGGCACTTGAAAGC |
| ZEB1 | PCR-F | GATGATGAATGCGAGTCAGATGC |
|  | PCR-R | ACAGCAGTGTCTTGTTGTTGT |
| Snail | PCR-F | ACTGCAACAAGGAATACCTCAG |
|  | PCR-R | GCACTGGTACTTCTTGACATCTG |

**Supplementary Table 3.** Clinicopathological information of 49 CCA patients.

| **Characteristics** | **Numbers (%)** |
| --- | --- |
| Numbers of patients | 49 |
| Average age (mean±SD) | 66.08±9.46 |
| Age (years) |  |
| <60 | 13 (26.53%) |
| ≥60 | 36 (73.47%) |
| Gender |  |
| Male | 26 (53.06%) |
| Female | 23 (46.94%) |
| Tumor site |  |
| Intrahepatic bile duct | 16 (32.65%) |
| Extrahepatic bile duct | 29 (59.18%) |
| Both | 4 (8.16%) |
| Histological grade |  |
| G1 | 3 (6.12%) |
| G2 | 27 (55.10%) |
| G3 | 13 (26.53%) |
| GX | 6 (12.24%) |
| Stage |  |
| I | 4 (8.16%) |
| II | 20 (40.82%) |
| III | 13 (26.53%) |
| IV | 4 (8.16%) |
| Missing | 8 (16.33%) |

**Supplementary Figure 1. DCLK1 activates PI3K/AKT/mTOR pathway in RBE.**

**
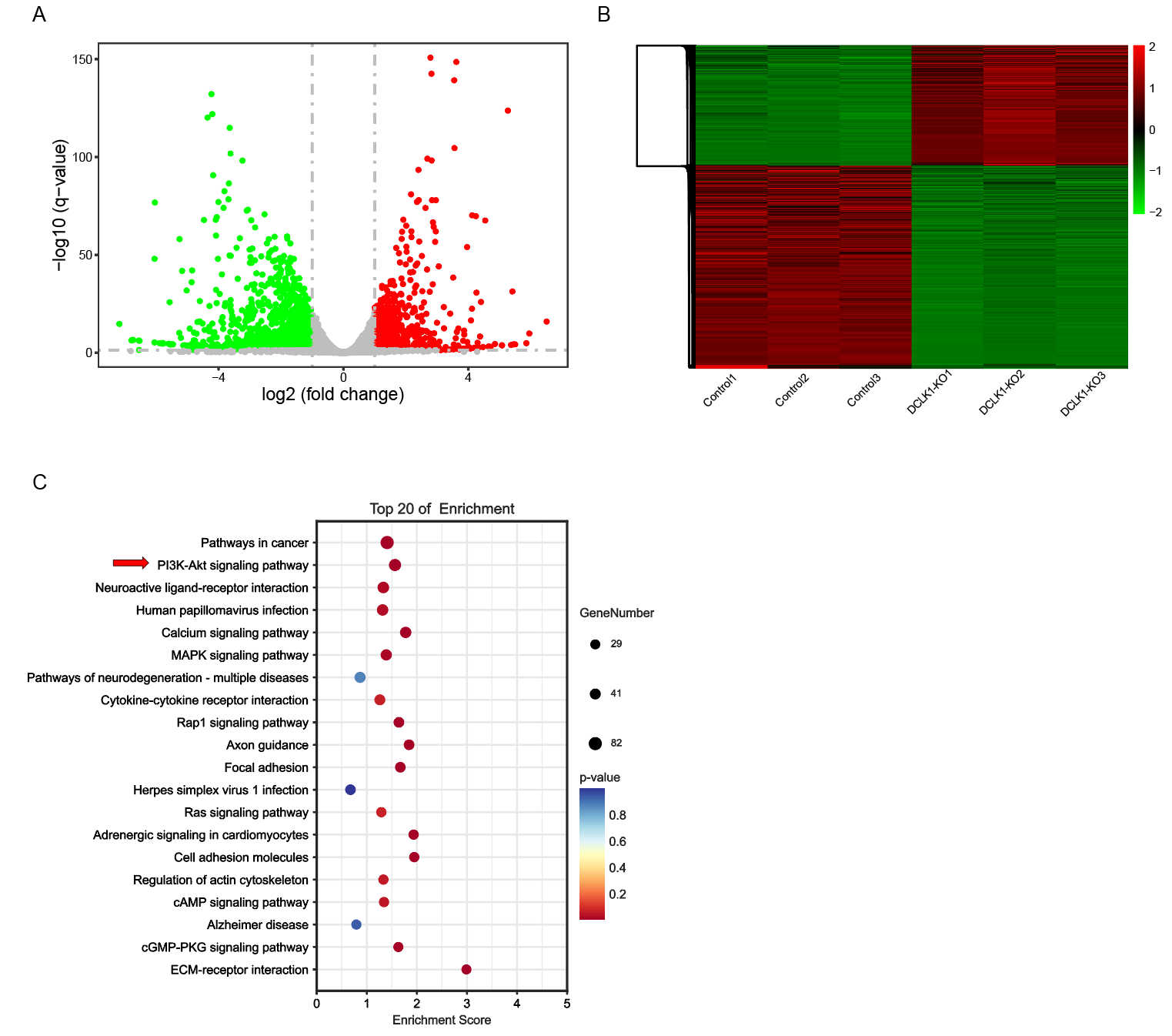
**

A. Volcano map of Different expressed genes (DEGs) between RBE control and RBE DCLK1-KO; B. Heatmap analysis of RBE control and RBE DCLK1-KO; C. KEGG analysis of DEGs screens the PI3K/AKT pathway as the key pathway related with DCLK1;
