# Supplementary figures and images for: Targeting the PI3K/AKT/mTOR pathway offer a promising therapeutic strategy for cholangiocarcinoma patients with high doublecortin-like kinase 1 expression
Source: J Cancer Res Clin Oncol. 2024 Jul 9;150(7):342. doi: 10.1007/s00432-024-05875-3 (PMC11233391; doi:10.1007/s00432-024-05875-3)

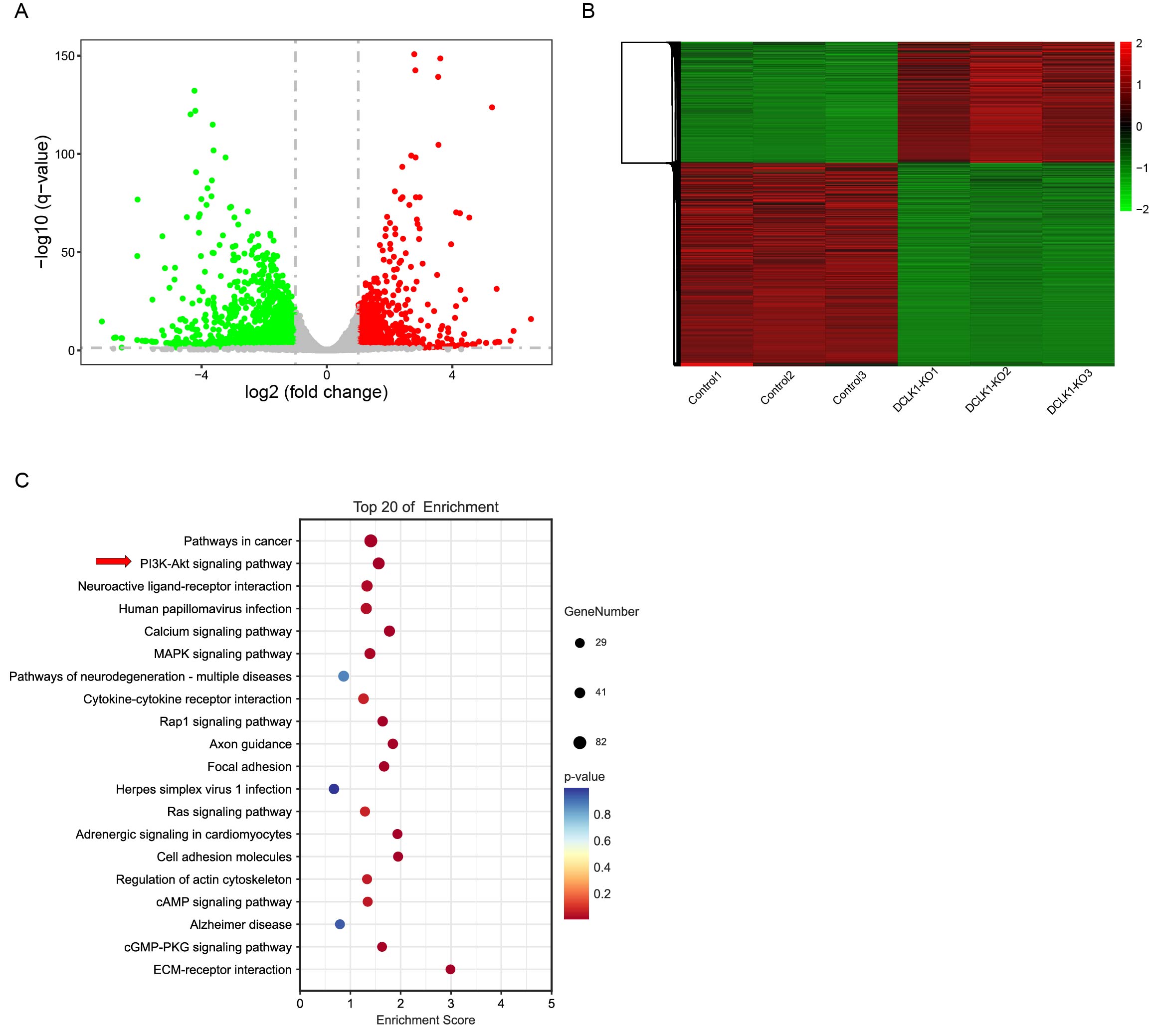

Supplement: Supplementary file 2 — Supplementary Figure 1. DCLK1 activates PI3K/AKT/mTOR pathway in RBE. A. Volcano map of Different expressed genes (DEGs) between RBE control and RBE DCLK1-KO; B. Heatmap analysis of RBE control and RBE DCLK1-KO; C. KEGG analysis of DEGs screens the PI3K/AKT pathway as the key pathway related with DCLK1 (JPG 307 KB) [file 432_2024_5875_MOESM2_ESM.jpg]
